# Effects of Phenyllactic Acid, Lactic Acid Bacteria, and Their Mixture on Fermentation Characteristics and Microbial Community Composition of Timothy Silage

**DOI:** 10.3389/fmicb.2021.743433

**Published:** 2021-12-16

**Authors:** Ping Li, Yongxiang Lu, Man Zhao, Liangyin Chen, Changbin Zhang, Qiming Cheng, Chao Chen

**Affiliations:** ^1^College of Animal Science, Guizhou University, Guiyang, China; ^2^Sichuan Academy of Grassland Sciences, Chengdu, China

**Keywords:** timothy silage, fermentation profile, phenyllactic acid, microbial community, high-throughput sequencing

## Abstract

This study investigated the effects of phenyllactic acid (PL), lactic acid bacteria (LAB), and their mixture on fermentation characteristics and microbial community composition of timothy silage. Timothy silages were treated without (CK) or with PL [10 mg/kg fresh matter (FM) basis], LAB inoculant (IN; a mixture of *Lactobacillus plantarum* and *L.buchneri*, 10^5^ cfu/g FM), and their mixture (PI) and stored at ambient temperature (5°C∼15°C) in a dark room for 60 days. Compared with CK, all treated silages showed lower (*P* < 0.05) levels of butyric acid and ammonia-N. Treatment with PL enhanced (*P* < 0.05) the crude protein preservation of silage by favoring the growth of *L. curvatus* and *Saccharomyces cerevisiae* and inhibition of lactic acid–assimilating yeast belonging to *Issatchenkia* during ensiling. In particular, treatment with PL advanced (*P* < 0.05) the productions of lactic acid and volatile fatty acid in IN-treated silage. Therefore, PL used as a new additive exhibited potential for improving silage fermentation when it is combined with LAB IN during ensiling.

## Introduction

Timothy (*Phleum pratense* L.) is one of the most important cool season grasses grown mainly in cold regions of North America, Scandinavia, Russia, and Japan (Berg et al., 1996) and usually used for pasture, hay, and silages (Bélanger et al., 2001). On the basis of the high total annual yield and good feeding value, timothy is also introduced and cultivated on the Qinghai Tibetan Plateau. However, how to effectively preserve the nutrient of timothy silage is a challenge for local livestock producer in this region.

Under anaerobic condition, lactic acid bacteria (LAB) dominate the fermentation process for a sufficient pH decline from the productions of organic acids [mainly lactic acid (LA)] to preserve the nutrients of forage. In practice, many factors such as low ambient temperature and packing density lead to incomplete or poor silage fermentation on the Qinghai Tibetan Plateau (Li et al., 2019). Under this condition, some undesirable microorganisms such as lactate-assimilating yeasts, low-temperature–resistant bacteria, and/or clostridia robust and reduce the stability of silage, resulting in high dry matter (DM) and economic losses. Biological additives are available for improving the fermentation quality of silage in the cold region (Chen et al., 2020a). However, the variable effectiveness from year to year is one of the main issues of using microbial inoculants (INs), because they are dependent on the environmental conditions and the forage characteristics (Muck et al., 2018). Therefore, chemical additives may be more effective in enhancing preservation of silage. Acids and/or their salts are the common active ingredients in chemical additives. The main acids used for silage additives are sorbic, benzoic, propionic, and acetic acids or their mixtures (Bernardes et al., 2015; Weiss et al., 2016). Commercial additives often contain mixtures of different acids at various concentrations to achieve the maximum effect against spoilage bacteria and fungi (Muck et al., 2018). Recently, some antimicrobial agents such as monopropionine and monobutyrin also showed a positive effect on silage preservation (Ferrero et al., 2019). In particular, propionate precursors such as fumaric, malic, citric, and succinic acids in the succinate-propionate pathway are applied in silage making (Guo et al., 2020).

Phenyllactic acid (PL) is an important broad-spectrum antimicrobial compound that inhibits the growth of undesirable microbes (most bacteria and some fungi) through multifaceted actions, even under low-temperature conditions (Rajanikar et al., 2021). PL is mainly derived from LAB genera such as *Lactobacillus*, *Pediococcus*, and *Weissella* (Bustos et al., 2018; Lipinska-Zubrycka et al., 2020; Wu et al., 2020). PL has been used in various bio-based materials in agricultural, pharmaceutical, and chemistry fields (Mu et al., 2009). In fact, PL is now well accepted as an alternative to antibiotics in livestock feeds (Nazareth et al., 2020). A study from Wu et al. (2020) has shown that PL could prevent crude protein (CP) degradation of alfalfa silage. However, limited information is available on how PL regulates microbiota for better silage preservation.

Hence, the aim of this study was to evaluate the effects of PL, LAB PL, and their mixture on the fermentation characteristics and microbial community composition of timothy silage on the Qinghai Tibetan Plateau. We hypothesized that PL was helpful for growth of LAB species during ensiling and subsequently advances silage fermentation of timothy in the cold region.

## Materials and Methods

### Silage Preparation

This study was conducted on the Hongyuan Experimental Base of Sichuan Academy of Grassland Sciences (N 31°51′–33°33′, E 101°51′–103°22′, altitude 3,500 m, Hongyuan, P.R. China). Timothy was harvested at the heading stage, chopped at a length of 1–3 cm, and divided into 12 equal piles. In total, 3 of 12 piles were randomly assigned to one of the following treatments: (i) no additive as control (CK); (ii) PL [L815533, provided from Macklin Biochemical Technology Co., Ltd., Shanghai, China; at an optimal application rate of 10 mg/L fresh matter (FM) basis]; (iii) LAB IN (a mixture of *Lactobacillus plantarum* and *L. buchneri*, provided from Gaofuji Biological Technology Co., Ltd., Chengdu, China; at a recommended application rate of 10^5^ cfu/g FM); (iv) PL + IN (PI). The LAB PL was diluted in sterilized water and applied using a hand sprayer, at a rate of 5 ml/kg of forage, by spraying uniformly onto the forage, which was constantly hand mixed. The PL was applied in a 500:1 w/w water-to-additive solution, using a hand sprayer, by spraying uniformly onto the forage, which was constantly hand mixed. The same amount of water was added to the CK treatment. The treated forage (about 50 kg) from each pile was divided into five equal parts. Each part (about 10 kg) was packed into a 20-L plastic bucket silo and sealed with a rubber gasket lid. All silages (*n* = 4 treatments × 5 storage time × 3 replications = 60) were stored in a dark room at ambient temperature (5∼15°C). Each treated silage was sampled after storage of 3, 7, 15, 30, and 60 days for determining the chemical composition and microbial community.

### Chemical Analysis

Frozen samples of 20 g were mixed with 180 ml of distilled water for 3 min in a Stomacher blender. The pH of the filtrate was determined by a pH meter (PHSJ-4F, Shanghai INESA Scientific Instrument Co., Ltd., Shanghai, China). Filtrate of about 10 ml was subjected to centrifugation (4,500 × *g*, 15 min, 4°C), and the supernatant was analyzed for LA, acetic acid (AA), propionic acid (PA), and butyric acid (BA) using high-performance liquid chromatography (Li et al., 2019). Ammonia-N was determined by the method of Broderick and Kang (1980).

The DM content of each sample from silage at 60 days was determined by oven drying at 65°C for 48 h. Dried samples were ground through a 1 mm screen with a mill (DFY-300C, Linda Machinery Co., Ltd., Wenling, China). The CP of each sample was analyzed by the Kjeldahl method (AOAC, 1990). Neutral detergent fiber (aNDF) and acid detergent fiber (ADF) were determined by the methods of Van Soest et al. (1991), using an fiber analyzer (ANKOM Technology, Fairport, NY), and expressed on a DM basis, including residual ash. When aNDF was measured, a heat-stable amylase (FAA, ANKOM Technology, Macedon, NY) was added following the instructions of the manufacturer. Water soluble carbohydrate (WSC) was determined by the method of Murphy (1958).

### Microbial Analysis

The microbial population was determined by the method of Cai et al. (1999). Ten grams of each moist sample was put into a sterile glass bottle, suspended in 90 ml of saline solution, and shaken for 45 min in a laboratory blender (LB20ES, Shanghai Primesci Co., Ltd., Shanghai, China). Serial dilutions from 10^–3^ to 10^–5^ were produced. LAB were counted on (de Man Rogosa an Medium) MRS agar (GCM188, Land Bridge Technology Co., Ltd., Beijing, China). Plates of LAB were incubated at 37°C for 48 h in the anaerobic box (TEHER Hard Anaerobox, ANX-1; Hirosawa Ltd., Tokyo, Japan). Molds and yeasts were counted on Rose Bengal Agar with tetracycline (1.5 mg/L; YM01435, Shyuanmu Biomart Biotech Co., Ltd., Shanghai, China) and incubated at 30°C for 72 h. Yeasts were distinguished from molds on the basis of colony appearance and cell morphology.

Total bacterial DNA from each sample of silages was extracted using cetyltrimethyl ammonium bromide (CTAB) method. The PCR amplification and bioinformatic analysis of samples were performed by the Novogene Bioinformatics Technology Co., Ltd., The primer pair 27F (5′-AGAGTTTGATCCTGGCTCAG-3′) and 1514R (5′- GNTACCTTGTTACGACTT-3′) were used to amplify the full-length 16S ribosomal RNA (rRNA) gene of the bacterial community. The primers (ITS9munngs TACACACCGCCCGTCG; ITS4ngsUni CCTSCSCTTANTDATATGC) were used to amplify the full-length ITS gene of the fungal community. The PCR reaction was conducted according to the description of Chen et al. (2020b). The purified PCR products were subject to generate libraries using SMRTbell™ Template Pre Kit (PacBio). The high-quality libraries were sequenced on the PacBio Sequel platform. The quality filter, cluster, and analysis for 16S rRNA and internally transcribed spacer (ITS) sequencing data were performed as described by Chen et al. (2020b) and Pootakham et al. (2021). The data were analyzed on Novogene Magic Platform.^1^

### Statistical Analysis

Data from silages were analyzed using the mixed procedure in SAS (v. 9.2). The following statistical model was applied: Y_*ij*_ = μ + S_*i*_ + I_*j*_ + S × I_*ij*_ + e_*ijk*_, where the fixed effects were as follows: μ = overall mean, S_*i*_ = additive treatment, I_*j*_ = storage period, S × I_*ij*_ = interaction between additive treatment and storage period, and e_*ij*_ = error. The differences between means were assessed by Tukey’s multiple comparison. The effect was considered significant when the probability was less than 0.05.

## Results

### Chemical Composition, Fermentation Profile, and Microbial Population of Silages

The chemical composition of silage is shown in Table 1. Compared with CK, treatment with PL increased (*P* < 0.05) WSC content and decreased the NDF content of silage. The highest CP content and the lowest NDF content were observed in PI-treated silages. All additives increased (*P* < 0.05) LAB count and decreased (*P* < 0.05) yeast number of silage as compared with CK.

**TABLE 1:** Chemical composition and microbial population of fresh forage and silage on the 60th day.

Treatments	DM^II^	WSC	CP	aNDF	ADF	LAB	Yeasts	Molds
			
	% FW	%DM				Log_10_ cfu/g FM		
Fresh forage								
	26.43	8.61	11.23	57.59	34.06	2.78	4.33	3.06
Silage^I^								
CK	24.43	0.59*^b^*	8.66*^b^*	68.88*^a^*	38.19	6.67*^d^*	5.21*^a^*	< 2.0
PL	24.31	1.52*^a^*	8.82*^b^*	64.11*^bc^*	38.06	8.00*^c^*	3.37*^b^*	<2.0
IN	25.15	0.71*^b^*	8.94*^b^*	67.29*^ab^*	37.13	8.95*^a^*	3.15*^c^*	< 2.0
PI	24.60	0.67*^b^*	10.46*^a^*	61.53*^c^*	36.70	8.34*^b^*	2.94*^c^*	<2.0
SEM	0.19	0.12	0.23	0.71	0.40	0.02	0.21	< 2.0
*P*-value	0.445	< 0.001	<0.001	0.007	0.539	<0.03	<0.001	—

*^a–c^Means within a row differed at level of P < 0.05.*

*^I^Silages were treated without (CK) or with phenyllatic acid (PL; at an optimal application rate of 10 mg/kg FM), lactic acid bacteria inoculant (IN; a mixture of L. plantarm and L. buchneri, at a recommended rate of 10^5^ cfu/g FM), and their mixture (PI).*

*^II^ADF, acid detergent fiber; CP, crude protein; DM, dry matter; FM, fresh matter; LAB, lactic acid bacteria; NDF, neutral detergent fiber; WSC, water-soluble carbohydrates; SEM, standard error of the mean.*

**FIGURE 1:**
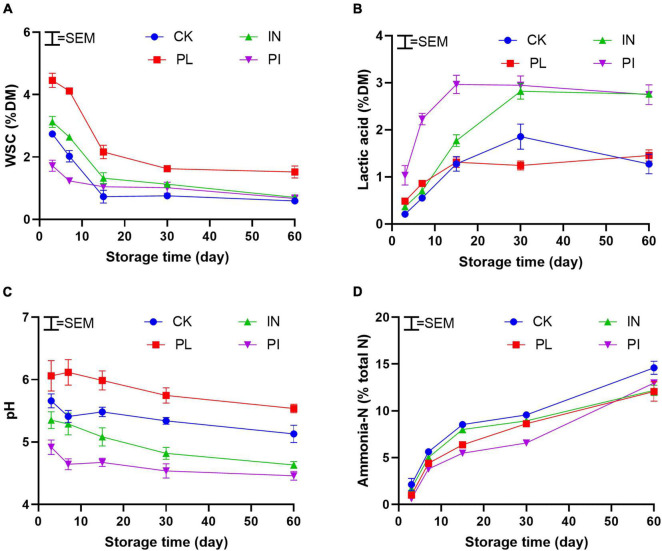
Dynamic of WSC **(A)**, lactic acid **(B)**, pH **(C)**, and butyric acid **(D)** of timothy silage during ensiling. Silages were treated without (CK) or with phenyllatic acid (PL; at an optimal application rate of 10 mg/kg FM), lactic acid bacteria inoculant (IN; a mixture of *L. plantarm* and *L. buchneri*, at a recommended rate of 10^5^ cfu/g FM), and their mixture (PI). SEM, standard error of mean.

**FIGURE 2:**
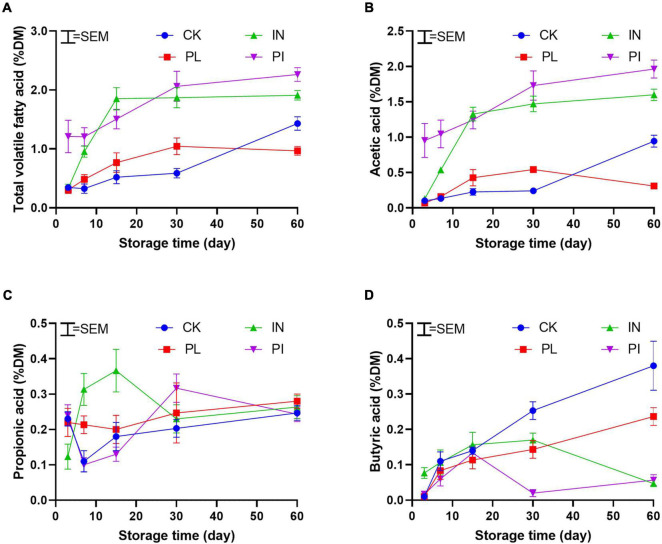
Dynamic of total volatile fatty acid **(A)** including acetic acid **(B)**, propionic acid **(C)**, and butyric acid **(D)** of timothy silage during ensiling. Silages were treated without (CK) or with phenyllatic acid (PL; at an optimal application rate of 10 mg/kg FM), lactic acid bacteria inoculant (IN; a mixture of *L. plantarm* and *L. buchneri*, at a recommended rate of 10^5^ cfu/g FM), and their mixture (PI). SEM, standard error of the mean.

As shown in Figure 1, treatment with PL delayed (*P* < 0.05) the WSC consumption, whereas treatment with IN and PI advanced LA production to reduce the pH value during the first 30 days of ensiling. All treated silages showed a delayed ammonia-N production, especially for PI-treated silage. Treatments with IN and PI promoted the production of total volatile fatty acid during ensiling (Figure 2). Compared with CK, treatment with PL decreased (*P* < 0.05) the AA content of silage on the 60th day. In contrast to CK- and PL-treated silages, the IN- and PI-treated silages showed a decreasing trend in BA after 15 days of ensiling.

### Bacterial and Fungal Alpha Diversities in Silage

The bacterial alpha diversity of silage is shown in Table 2. The observed species, ACE, and Shannon firstly increased and then decreased with prolonged ensilage time in CK silage. An opposite trend occurred to PL-and IN-treated silages. Compared with CK, additives increased bacterial alpha diversity indices of silage on the 7th day but decreased that of silage on the 30th day. In relative to PL-and IN-treated silages, low bacterial alpha diversity indices occurred to the PI-treated silage.

**TABLE 2:** The alpha-diversity of bacterial community of silage samples.

Samples^I^	PE reads	Observed species	ACE	Shannon	Coverage
CK7^II^	6,244	21	21.45	0.58	0.999
CK30	10,616	50	54.71	2.73	0.993
CK60	12,689	31	32.05	1.23	0.993
PL7	11,563	43	46.54	1.74	0.983
PL30	4,668	24	24.43	0.68	0.996
PL60	7,929	32	40.22	1.19	0.989
IN7	11,623	36	48.16	1.39	0.997
IN30	14,085	32	33.59	0.81	0.992
IN60	11,601	39	40.02	2.30	0.994
PI7	12,114	33	33.44	0.83	0.992
PI30	12,536	25	29.48	1.10	0.996
PI60	9,899	16	21.19	0.43	0.996

*^I^Samples from silages treated without (CK) or with phenyllatic acid (PL; at an optimal application rate of 10 mg/kg FM), lactic acid bacteria inoculant (IN; a mixture of L. plantarm and L. buchneri, at a recommended rate of 10^5^ cfu/g FM), and their mixture (PI).*

*^II^Silages were sampled at days 7, 30, and 60 of ensiling, respectively.*

The fungal alpha diversity of silage is shown in Table 3. The indices of ACE and Shannon were increasing in CK- and IN-treated silages during ensiling. No differences in the indices of observed species, ACE, and Shannon of silages on the 30th day were observed between the CK and PL treatment. Compared with CK, treatments with PL and PI decreased fungal alpha diversity indices of silage on the 60th day. Notably, the highest values of observed species, ACE, and Shannon were found in IN-treated silage on the 60th day.

**TABLE 3:** The alpha-diversity of the fungal community of silage samples.

Samples^I^	PE reads	Observed species	ACE	Shannon	Coverage
CK7^II^	8,546	21	29.53	1.62	0.997
CK30	9,506	50	65.23	2.85	0.992
CK60	9,522	50	88.60	3.00	0.989
PL7	10,363	27	74.78	1.79	0.993
PL30	8,323	50	62.06	2.91	0.992
PL60	9,609	33	65.12	2.16	0.992
IN7	5,718	30	43.86	1.55	0.997
IN30	6,663	45	59.58	1.79	0.992
IN60	3,913	117	174.19	3.78	0.977
PI7	8,372	29	38.62	1.69	0.996
PI30	9,068	34	64.73	2.43	0.992
PI60	8,371	43	44.75	2.45	0.998

*^—^Samples from silages treated without (CK) or with phenyllatic acid (PL; at an optimal application rate of 10 mg/kg FM), lactic acid bacteria inoculant (IN; a mixture of L. plantarm and L. buchneri, at a recommended rate of 10^5^ cfu/g FM), and their mixture (PI).*

*^II^Silages were sampled at days 7, 30, and 60 of ensiling, respectively.*

### Bacterial and Fungal Community Compositions in Silage

**FIGURE 3:**
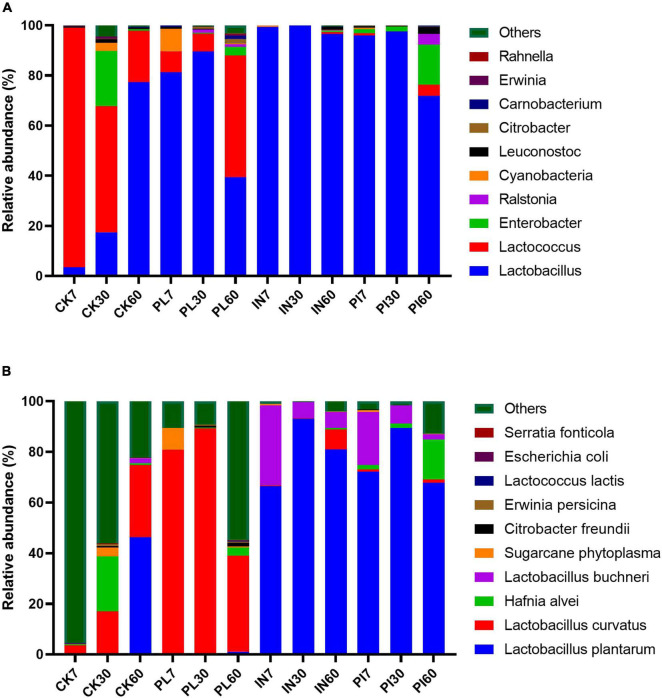
Relative abundance of top 10 genera **(A)** and species **(B)** of the bacterial community in timothy silage. Silages were treated without (CK) or with phenyllatic acid (PL; at an optimal application rate of 10 mg/kg FM), lactic acid bacteria inoculant (IN; a mixture of *L. plantarm* and *L. buchneri*, at a recommended rate of 10^5^ cfu/g FM), and their mixture (PI).

The bacterial community composition of silage is illustrated in Figure 3. *Lactobacillus*, *Lactococcus*, and *Enterbacter* were the top three genera, with a total relative abundance of > 90%. The abundance of *Lactobacillus* increased in the CK silage during ensiling. High proportions of *Lactococcus* existed in PL-treated silage on the 60th day. In addition, treatment with PI facilitated the increase in abundance of *Enterobacter* in silage during ensiling. At species level, *L. curvatus* dominated in CK- and PL-treated silages during the first 30 days of ensiling, whereas *L. plantarum* dominated in IN-and PI-treated silages during ensiling.

**FIGURE 4:**
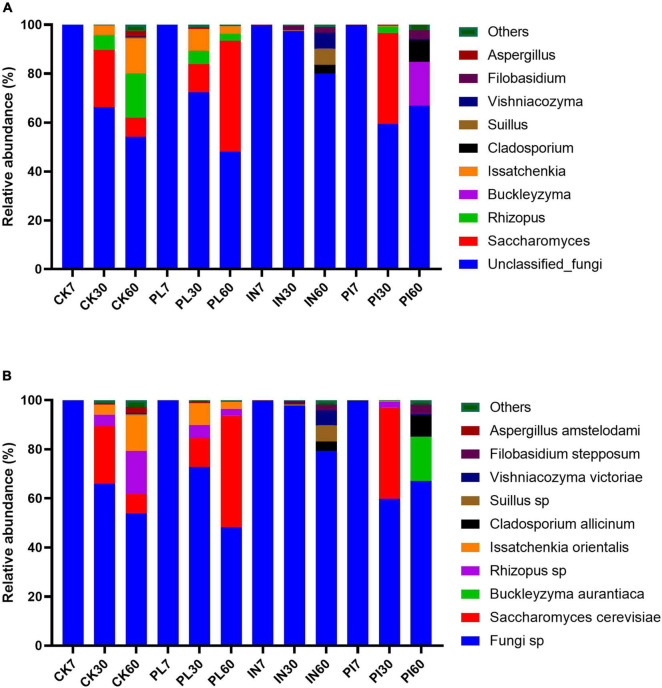
Relative abundance of top 10 genera **(A)** and species **(B)** of fungal community in timothy silage during ensiling. Silages were treated without (CK) or with phenyllatic acid (PL; at an optimal application rate of 10 mg/kg FM), lactic acid bacteria inoculant (IN; a mixture of *L. plantarm* and *L. buchneri*, at a recommended rate of 10^5^ cfu/g FM), and their mixture (PI).

The fungal community composition is illustrated in Figure 4. Most genera were unclassified fungi in silages. Prolonged ensilage increased abundance of *Saccharomyces*, *Rhizopus*, *Buckleyzyma*, *Issatchenkia*, and *Cladosporium allicinum*. Treatment with PL stirred the increase in abundance of *Saccharomyces* and decrease in *Rhizopus* and *Issatchenkia* in silage on the 60th day. At the species level, the abundance of *Rizopus* sp. and *Issatchenkia orientalis* increased in CK silage during ensiling. A similar trend occurred to *Saccharomyces cerevisiae* in PL-treated silage.

## Discussion

### Chemical Composition, Fermentation Profile, and Microbial Population of Silages

Silage fermentation is driven by microbes (mainly LAB) under anaerobic conditions. Available substrates such as WSC are important for the propagation and growth of desirable microorganisms. In the present study, the WSC concentration (Table 1) was sufficient for initiating silage fermentation. However, low LAB counts and amounts of detrimental yeasts on the fresh plant were not beneficial for silage preservation. In fact, the control silage showed poor fermentation characteristics with high levels of pH, ammonia-N, and BA (Figures 1, 2).

Various strategies have been used to reduce nutrient loss of silage. During ensiling, CP is degrading gradually, as indicated by increasing ammonia-N formation and BA production. Microbial PLs are recognized as important and widely used additives to improve the fermentation quality of silage. In the present study, however, the inoculation of LAB did not result in a high CP level in silage (Table 1). Our previous study has proved that the effectiveness of LAB inoculation varied between forage resources, management practice, and storage conditions (Chen et al., 2020a). PL has been applied in feed diets for improving animal performance. A study from Wu et al. (2020) has proved that PL exhibited a positive effect on the CP preservation of alfalfa silage. This situation was just observed in PI-treated silage. In addition, PL decreased the WSC consumption during ensiling, with a low fiber (NDF and ADF) concentration in silage. These indicated that a combination of PL and LAB could enhance the preservation of silage nutrients.

The preservation of silage nutrients was dependent on the fermentation rate and extent. The control silage exhibited a low production of LA and reduction of pH during silage fermentation (Figure 1). Most LAB PLs showed poor performance in silage production in cold regions because strains in commercial PLs are selected in temperate regions. In the present study, however, inoculation of LAB advanced the pH decline by quickly yielding LA during ensilage. A similar result was from Chen et al. (2020a,b) who reported that the use of LAB PL could advance the fermentation process of alfalfa and oat silages on the Qinghai Tibetan Plateau. AA is the active ingredient for controlling undesirable microorganisms such as yeasts in silage after exposure to air. Most commercial PLs contain hetero- and homo-fermentative LAB species, which could stabilize silage with the production of the most volatile fatty acids such as AA and PA. Thus, the AA in LAB-inoculated silage accumulated also at an early stage of ensiling (Figure 2). However, the AA concentration sustained stable after 30 days of ensiling. This may be attributable to how the species of AA-producing bacteria exhibited low activity under a low temperature of < 15°C (Zhou et al., 2016). PL is considered a natural environmentally friendly organic acid. The effect of PL on the fermentation characteristics of silage is controversial. Xu et al. (2019) found that PL had no significant effect on the fermentation products in whole-crop corn silage. A similar result was from Jung et al. (2019) who reported that PL level was not consistent with LA production in kimchi fermentation. Wu et al. (2020) used PL as an additive at ensiling and found that PL could promote the production of LA, AA, and PA in alfalfa silage. In the present study, the use of PL at ensiling did not enhance the fermentation performance of timothy silage. However, a synergistic effect of PL and LAB PL to effective advance productions of LA and total volatile fatty acid (mainly AA) for quick pH decline at an early stage of ensiling (Figures 1, 2). The behind mechanism is worth exploring.

### Bacterial and Fungal Alpha Diversities in Silage

As described previously, epiphytic microbiota regulated the fermentation rate and extent of silage. In turn, the microbial community was shaped by fermentation products. In the present study, the diversity (Shannon) and richness (ACE) indices of the bacterial community in control silage firstly increased and then decreased with the prolonged ensilage time (Table 2). Similar to our finding, an increase in bacterial diversity and richness indices occurred in barley, oat, triticale, and intercrop silages with relatively high pH levels (Duniere et al., 2017). However, the diversity and richness decline because of the continuous pH reduction from the accumulation of fermentation acids during ensiling (Polley et al., 2007). Ogunade et al. (2018) revealed that silage inoculated with LAB had decreased bacterial diversity indices due to the increased relative abundance of the predominant genus of *Lactobacillus*. This partly explained the decreased bacterial alpha-diversity of LAB-inoculated silage at the early stage of ensiling. However, the low temperature inhibited the fermentation of LAB-inoculated silage, resulting in a higher pH value, which provided conditions for undesirable microorganisms and subsequently increased the bacterial diversity (Chen et al., 2020b). The PL-treated silage showed a similar trend in bacterial alpha diversity. On the basis of weak acid theory, PL effectively inactivated most bacteria (Rajanikar et al., 2021), thus quickly reducing the diversity and richness of the bacterial community. When the silage continued to ferment for a lower pH value, the inhibitory effect was reduced, and the spoilage microorganisms robust again at the final stage of ensiling. Notably, a desirable situation in linearly decreasing diversity and richness of bacterial community during ensiling was found in PI-treated silage, indicating that the combinatory effect from PL and LAB was desirable.

Researchers pay limited attention to the fungal community in well-fermented silages, due to the absence of toxin-producing fungi (Duniere et al., 2017). The diversity and richness of the fungal community were increasing in CK silage during ensiling (Table 3). A similar result was found by Vu et al. (2019) who reported that the fungal community richness increased in elephant grass silage under natural conditions. Furthermore, this increasing trend was enhanced by the inoculation of LAB. Liu et al. (2019) reported that the fungal diversity and richness increased in LAB inoculated silage during ensiling. However, inconsistent results came from Keshri et al. (2018) who reported a remarkable decline in fungal Chao 1 index due to acidic and anaerobic conditions developed in *L. plantarum*–treated corn silage during ensiling. Research from Lavermicocca et al. (2003) demonstrated that the addition of LA could cause a 30% increase in the inhibitory activity of PL against fungi. Thus, the positive effect from the combination of PL and LAB on reducing diversity and richness of fungal community was observed in silage on the 60th day.

### Bacterial and Fungal Community Compositions in Silage

The diversity and richness of microbial community were negatively correlated with the dominance of functional genera or species. Various microbial communities and succession were found in different silages (Parvin et al., 2010), and it is necessary to know the microbial community compositions to understand the complex process of ensiling (Xu et al., 2019). In the present study, *Lactobacillus*, *Lactococcus*, and *Enterbacter* were the top three genera, with a total relative abundance of > 90% (Figure 3). Studies from Cai et al. (1998) reported that *cocci* such *Lactococcus* prevail at the early stage of ensiling and were replaced by low-pH–resistant rods such as *Lactobacillus* species in silage. This situation was confirmed well in the present study because the ratio of epiphytic *Lactobacillus*/*Lactococcus* in control silage was increasing as the ensilage time prolonged (Figure 3). Inoculation of LAB could enhance silage fermentation by dominating the *Lactobacillus*. PL also advanced the dominance of *Lactobacillus*, but this positive effect was reduced at the late stage of ensiling, with high proportions of *Lactococcus* in silage at 60th day. At the species level, *L. curvatus*, *L. plantarum*, and *L. buchneri* dominated in silage. *L. curvatus* is mainly present in fermented foods. In this study, PL facilitated the growth of *L. curvatus* and extensively promoted the disappearance of inherent *L. plantarum* in untreated silage. However, PL exerted a positive effect on the dominance of *L. plantarum* in IN-treated silage. The behind reason is unknown. In addition, the relative abundance of *L. buchneri* in LAB-treated silage showed a decreasing trend during storage. Similarly, Zhou et al. (2016) and Chen et al. (2020b) reported that *L. buchneri* was disappeared or undetectable in corn, oat, and alfalfa silages under low temperatures.

The dominant fungal genera in silage was unclassified fungi (Figure 4). This was not in accordance with other results. The majority of fungi reported by Liu et al. (2019) in barley silages were *Issatchenkia*, *Cladosporium*, and *Alternaria*. Bai et al. (2020) found that unclassified fungi *Sporormiaceae*, *Ascochyta*, and *Candida* dominated in untreated-alfalfa silage. Genera of *Kazachstania*, *Cadida*, and *Picha* were heavily presented in natural-fermented sugar top silage (Wang et al., 2020). Many factors such as forage types and ambient storage temperature resulted in the discrepancy in fungal community composition. During ensiling, the total abundance of *Saccharomyces*, *Rhizopus*, and *Issyatchenkia* was increasing in control and PL-treated silages; *Cladosporium*, *Suillus*, and *Vishniacozyma* in PI-treated silages; and *Buckleyzyma*, *Cladosporium*, and *Filobasidium* in PI-treated silages. This indicated that the use of additives stirred the changes in fungal community compositions. The high distribution of yeasts belonging to *Issyatchenkia* is an indicator for the low aerobic stability of silage. The succession of fungal species in silage upon aerobic exposure is typically initiated by yeasts with the increase in pH, thereby allowing the low number of acid-tolerant spoilage microorganisms to proliferate (McAllister et al., 1995). *Saccharomyces cerevisiae* is considered a non-spoilage yeast because it does not assimilate LA and prevails in ryegrass, oat, barley, and sugar top silages (McAllister et al., 2018). According to the fact that the high abundance of LA-assimilating *Issyatchenkia* species decreased the aerobic stability of silages after exposure to air (Liu et al., 2019), thereby the addition of phenyllatic acid in timothy silage exerted a positive response to delay aerobic deterioration evoked by the proliferation of spoilage fungi. *Cladosporium*, as a producer of mycotoxins, is a member of the *Davidiellaceae* family and a ubiquitous mold (Tabuc et al., 2011). The presence of *Cladosporium* indicated that inoculation of LAB showed a limited effect on the stability of silage at the final stage of ensiling. Genera of *Rhizopus* occurred rarely in silages (Rodríguez-Blanco et al., 2021). It was first reported that *Buckleyzyma salicina* distributed in timothy silage.

## Conclusion

Natural-fermented timothy silage showed poor fermentation on the Qinghai Tibetan Plateau. The use of PL could facilitate the growth of *L.* curvatus for better preservation of silage nutrients with low levels of ammonia-N and BA. The application of PL also showed a positive effect on the rapid production of LA and volatile fatty acid in LAB-inoculated silage. However, all silages exhibited potential for aerobic deterioration due to the high presence of undesirable fungi such as *Saccharomyces*, *Cladosporium*, and/or *Issatchenkia*. Further study is still needed to investigate the mechanism of reduced antifungal activity of PL during silage fermentation under low-temperature conditions.

## Data Availability Statement

The original contributions presented in the study are included in the article/supplementary material, further inquiries can be directed to the corresponding author/s.

## Author Contributions

PL and CC: conception and design of study and critical review and revision. YL, MZ, LC, and CZ: acquisition of data. PL and QC: analysis and interpretation of data. PL and YL: drafting the manuscript. QC and MZ: others. All authors read and contributed to the manuscript.

## Conflict of Interest

The authors declare that the research was conducted in the absence of any commercial or financial relationships that could be construed as a potential conflict of interest.

## Publisher’s Note

All claims expressed in this article are solely those of the authors and do not necessarily represent those of their affiliated organizations, or those of the publisher, the editors and the reviewers. Any product that may be evaluated in this article, or claim that may be made by its manufacturer, is not guaranteed or endorsed by the publisher.

## Footnotes

1
https://magic.novogene.com
